# Design of a Metasurface-Enhanced Mid-Infrared Biosensor for Fingerprint Signal Enhancement of *Staphylococcus aureus* Biofilms

**DOI:** 10.3390/bios16070397

**Published:** 2026-07-22

**Authors:** Bowei Yang, Ang Zhou, Yuxiang Yang, Yu Zhao, Chunying Pang

**Affiliations:** 1 School of Life Science and Technology, Changchun University of Science and Technology, No. 7186 Weixing Road, Changchun 130022, China; 2024800055@cust.edu.cn (B.Y.); 2020003109@mails.cust.edu.cn (A.Z.); 2 Postdoctoral Research Station of Instrument Science and Technology, Changchun University of Science and Technology, No. 7089 Weixing Road, Changchun 130022, China; 3 School of Optoelectronic Engineering, Changchun University of Science and Technology, No. 7186 Weixing Road, Changchun 130022, China; 2025200094@mails.cust.edu.cn; 4 Changchun Institute of Optics, Fine Mechanics and Physics, Chinese Academy of Sciences, No. 3888 Dong Nanhu Road, Changchun 130033, China; zhaoyu@ciomp.ac.cn

**Keywords:** optical biosensor, surface-enhanced infrared absorption (SEIRA), metasurface, mid-infrared spectroscopy, bacterial biofilm, *Staphylococcus aureus*

## Abstract

Mid-infrared spectroscopy provides molecular fingerprint information for bacterial biofilm analysis, but the absorption signal of a thin biofilm layer is usually weak. In this work, a metasurface-enhanced mid-infrared biosensor was designed to enhance the fingerprint response of *Staphylococcus aureus* biofilms. The biofilm transmission spectrum was measured by Fourier-transform infrared spectroscopy, and the film thickness was obtained by atomic force microscopy using an edge step-height method. Based on these measurements, an effective extinction coefficient was extracted and used in finite-difference time-domain simulations. A metal–insulator–metal metasurface was then optimized to cover the main biofilm absorption bands in the mid-infrared region. Two resonator designs were studied: a polarization-dependent structure and a polarization-insensitive structure. The polarization-dependent design showed a strong response under x-polarized incidence and weak coupling under y-polarized incidence. The polarization-insensitive design provided a more balanced response for orthogonal polarizations. At the selected biofilm fingerprint wavelengths, the highest enhancement factors reached 8.57 and 7.24 for the polarization-dependent and polarization-insensitive structures, respectively. Near-field distributions confirmed that the enhancement mainly originated from localized electric fields at the metal resonator edges. These results provide a proof-of-concept design strategy for enhancing weak mid-infrared fingerprint signals from *S. aureus* biofilms.

## 1. Introduction

Mid-infrared spectroscopy is a useful label-free method for biological analysis. Molecular vibrations in this region provide fingerprint information from proteins, lipids, polysaccharides, nucleic acids, and cell-wall components. Therefore, FTIR spectroscopy has been widely used in microbial cell analysis, environmental microbiology, and bacterial identification [1]. It has also been applied to the analysis of *Staphylococcus aureus* cell-wall chemical features [2]. Bacterial biofilms are surface-attached microbial communities embedded in extracellular polymeric substances. The biofilm matrix contains water, polysaccharides, proteins, lipids, and extracellular DNA [3]. *S. aureus* can form stable biofilms on different surfaces, which makes it more persistent and harder to remove [4,5]. Sensitive detection of *S. aureus* biofilms is therefore important for surface contamination analysis, public health, and environmental monitoring. Optical and photonic biosensors provide label-free and non-destructive readout for biological targets and have been widely investigated for healthcare, environmental monitoring, and food/water quality control. Nanomaterial- and metasurface-mediated optical transducers can improve molecular detectability and extend biosensing platforms across broad spectral regions, including the infrared and terahertz ranges [6]. For pathogen-related monitoring, biosensors are attractive because they can provide rapid, sensitive, and potentially on-site analysis [7]. However, the infrared absorption signal from a thin biofilm layer is usually weak. This limits the direct use of conventional infrared spectroscopy when the amount of biofilm is small. This issue is also important for monitoring biofilm formation, where a non-destructive method with higher spectral sensitivity is useful [8].

Surface-enhanced infrared absorption (SEIRA) spectroscopy can improve the sensitivity of infrared detection. In SEIRA, a resonant structure confines the electromagnetic field near the sensing surface. When the target material is located in this near-field region, its vibrational absorption can be enhanced [9,10]. The first enhanced infrared absorption from molecular layers on metal films was reported several decades ago [11]. Later studies showed that the SEIRA response depends on the resonator geometry, resonance position, scattering behavior, and local field distribution [12,13,14]. Therefore, the resonator should be designed according to the target absorption bands, rather than only the resonance of the bare structure. SEIRA has been applied to many chemical and biological targets. Resonant plasmon-enhanced SEIRA has been used to detect and discriminate mixed sugar solutions [15]. Large-area nanorod arrays have also been developed to obtain stable SEIRA responses over macroscopic areas [16]. For microbial analysis, gold nanorod arrays have been used to collect enhanced infrared spectra of *Escherichia coli*, *S. aureus*, and *Bacillus subtilis* [17]. Silver nanoparticle substrates have also been used for SEIRAS-based discrimination of microorganisms, including *S. aureus* [18]. In addition, nanostructured silicon surfaces and mid-infrared resonant silicon microstructures have been reported for enhanced bacterial infrared detection [19,20]. In addition to SEIRA, other surface-enhanced vibrational spectroscopy techniques have also been investigated for biological sensing. For example, surface-enhanced Raman spectroscopy (SERS) based on nanostructured substrates has been used for biological sample detection and discrimination [21]. These studies show the potential of surface-enhanced vibrational spectroscopy for biological sensing. However, studies focused on the broad mid-infrared fingerprint enhancement of *S. aureus* biofilm are still limited. In particular, it is useful to design metasurface structures that can enhance several characteristic absorption bands of the biofilm in the same spectral region.

In this work, we design a metasurface-enhanced mid-infrared biosensor for fingerprint-signal enhancement of *S. aureus* biofilms. The biofilm transmission spectrum and thickness are measured by FTIR and AFM, and the effective extinction coefficient is then obtained for optical modeling. Based on the main absorption bands of the biofilm, two metasurface structures are designed: a polarization-dependent structure and a polarization-insensitive structure. Their spectral responses, enhancement factors, and near-field distributions are evaluated using FDTD simulations. The results provide a biosensor design route for enhancing weak mid-infrared fingerprint signals from thin *S. aureus* biofilms.

## 2. Optical Parameter Extraction of *Staphylococcus aureus* Biofilm

**Figure 1:**
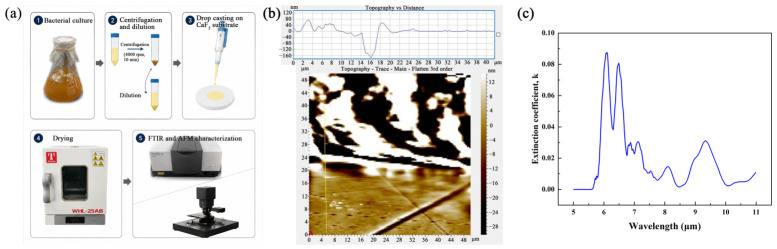
Optical parameter extraction of the *Staphylococcus aureus* biofilm. (**a**) Workflow of biofilm preparation and characterization. (**b**) AFM image and edge height profile of the dried biofilm; the yellow line indicates the path used to extract the height profile. (**c**) Extracted extinction coefficient k of the biofilm.

The optical parameters of the *Staphylococcus aureus* biofilm were extracted before the metasurface simulation. The procedure is shown in Figure 1. A 200 μL bacterial suspension was dropped onto a CaF_2_ substrate with a diameter of 2 cm. The sample was then dried at 60 °C to form a biofilm layer. After drying, the sample was characterized by FTIR and AFM. The infrared transmission spectrum was measured using the CaF_2_ substrate as the reference. The measured transmission data were used to estimate the effective loss of the dried biofilm. The biofilm thickness was obtained from the AFM image by an edge step-height method. Since the AFM scan area was limited to 50 × 50 μm^2^, an edge region was selected to measure the local height difference between the biofilm-covered area and the exposed substrate. The effective thickness used in the optical model was set to 80 nm.

The deposited biofilm was not fully uniform. It contained bacterial cells, extracellular components, pores, and possible residual water. Therefore, it was treated as an effective thin film rather than a homogeneous bulk material. This treatment is reasonable because the infrared wavelengths used in this work are much larger than the biofilm thickness. For example, the target wavelengths are in the range of about 5–11 μm, while the modeled film thickness is only 80 nm. In this long-wavelength limit, the incident wave mainly probes the averaged optical response of the thin biofilm layer. Thus, the biofilm can be described by an effective complex refractive index:

(1) $$\tilde{n}(\lambda )=n+ik(\lambda )$$

where n is the real part of the refractive index and k is the extinction coefficient. The real part of the refractive index was set to n = 1.4. This value is consistent with previous optical studies, where the refractive index of biofilms was found to be close to that of water, and an effective biofilm medium with n = 1.4 was used in plasmonic optical modeling [22,23]. The extinction coefficient k was calculated from the measured transmission and the AFM-derived thickness. The normalized transmission through the effective biofilm layer can be described as follows:

(2) T(λ)=exp[−α(λ)d]

where T(λ) is the normalized transmission, α(λ) is the absorption coefficient, and d is the effective biofilm thickness. Thus,

(3) $$\alpha (\lambda )=-\frac{\ln T(\lambda )}{d}$$

The extinction coefficient was then obtained from the following:

(4) $$k(\lambda )=\frac{\alpha (\lambda )\lambda }{4\pi }$$

The extracted extinction coefficient is shown in Figure 1c. Several characteristic absorption features can be observed near 6.06, 6.49, 8.13, 9.26, and 9.71 μm, which may be tentatively attributed to protein amide vibrations as well as carbohydrate- and phosphate-related components in bacterial cells and extracellular polymeric substances. These characteristic bands were selected as the target wavelengths for the subsequent SEIRA metasurface design.

## 3. Metasurface Parameter Optimization

A metal–insulator–metal metasurface was designed to enhance the mid-infrared absorption of the biofilm. The structure consisted of top Au resonators, a CaF_2_ spacer, and a bottom Au layer. The bottom Au layer suppressed transmission, so the optical response was mainly evaluated by reflectance.

The finite-difference time-domain (FDTD) simulations were performed using the commercial software ANSYS FDTD Solutions 2024a. A broadband plane-wave source was used to illuminate the structure under normal incidence. The reflectance spectra were obtained using frequency-domain power monitors placed above the metasurface. The mesh size was set to Δx = Δy = 0.05 μm and Δz = 0.02 μm to resolve the metallic resonators, dielectric spacer, and thin biofilm layer. Periodic boundary conditions were used in the in-plane directions, and perfectly matched layers were used along the propagation direction. In this step, an 80 nm dielectric layer with n = 1.4 and k = 0 was placed on the metasurface. This lossless biofilm layer was used as a reference layer. It allowed the refractive-index loading from the biofilm to be included during the geometric optimization, while excluding the absorption loss from k. This is important because adding the biofilm changes the local dielectric environment and can shift the resonance peak.

**Figure 2:**
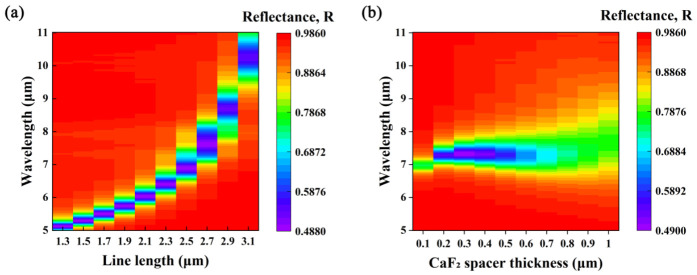
Parameter optimization of the metasurface. (**a**) Reflectance map as a function of Au line length. (**b**) Reflectance map as a function of CaF_2_ spacer thickness.

Two geometric parameters were scanned: the length of the Au strip and the thickness of the CaF_2_ spacer. Figure 2a shows the reflectance map as a function of the Au strip length. The resonance shifts to longer wavelengths as the strip length increases. This trend indicates that the resonance position can be tuned by changing the effective resonator length. The resonance can therefore be adjusted to overlap with the main absorption bands of the biofilm.

Figure 2b shows the reflectance map as a function of the CaF_2_ spacer thickness. The spacer thickness changes the coupling between the top Au resonator and the bottom Au layer. It also affects the resonance width and the reflectance level. In this work, the target was not to design a perfect absorber. For SEIRA sensing, the enhanced signal comes from the interaction between the localized field and the biofilm absorption. If the original resonance is too deep or too narrow, the spectrum may leave little room for the additional biofilm absorption to be resolved. Therefore, a broader resonance with moderate absorption was preferred. Based on this consideration, the CaF_2_ spacer thickness was selected as t = 1 μm.

The parameter scans show that the MIM metasurface can provide tunable resonances in the 5–11 μm range after refractive-index loading by the biofilm layer. This range covers the main absorption bands extracted in Figure 1. Based on this design rule, two metasurface structures were further studied. The first one was a polarization-dependent structure, which gives a strong response under x-polarized incidence and a weak response under y-polarized incidence. The second one was a polarization-insensitive structure, which gives similar responses for the two orthogonal polarizations.

## 4. Polarization-Dependent SEIRA Metasurface

**Figure 3:**
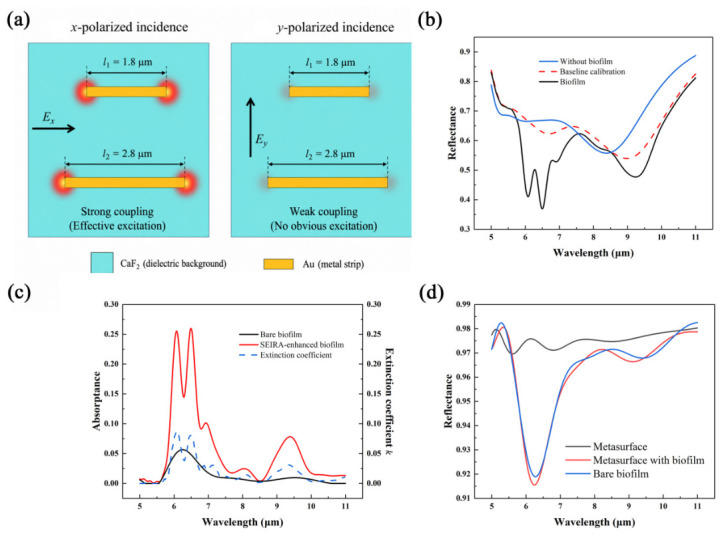
Polarization-dependent metasurface and spectral response. (**a**) Top-view schematic of the polarization-dependent resonator. The red regions indicate the locations of strong electric-field oscillations in the resonator. (**b**) Reflectance spectra under x-polarized incidence. (**c**) Bare and SEIRA-enhanced biofilm absorption spectra. (**d**) Reflectance spectra under y-polarized incidence.

The first metasurface was designed as a polarization-dependent structure. As shown in Figure 3a, the top resonator consists of two horizontal Au strips with different lengths. The strip lengths were set as l_1_ = 1.8 μm and l_2_ = 2.8 μm. This asymmetric resonator mainly couples with the electric field along the x direction. Therefore, a strong response is expected under x-polarized incidence, while a weak response is expected under y-polarized incidence.

The reflectance spectra under x-polarized incidence are shown in Figure 3b. Three cases were simulated: the metasurface without biofilm, the metasurface with baseline calibration, and the metasurface with biofilm. For baseline calibration, an 80 nm biofilm layer with n = 1.4 and k = 0 was placed on the metasurface. This baseline includes the refractive-index loading of the biofilm but excludes the absorption loss. Thus, it can be used to separate the absorption contribution of the biofilm from the resonance shift caused by the real refractive index.

The SEIRA-enhanced absorption signal was calculated by subtracting the biofilm-covered spectrum from the baseline-calibrated spectrum:

(5) $$A_{\mathrm{SEIRA}}\left(\lambda \right)\;=\;R_{\mathrm{baseline}}\left(\lambda \right)\;-{\;R}_{\mathrm{biofilm}}\left(\lambda \right)$$

where R_baseline_ is the reflectance of the metasurface after baseline calibration, and R_biofilm_ is the reflectance of the metasurface covered by the biofilm with the extracted k. The calculated result is shown in Figure 3c. For comparison, the absorption of the bare biofilm and the extracted extinction coefficient k are also plotted. The enhanced absorption spectrum follows the main absorption features of the biofilm, especially at 6.06, 6.49, 8.13, 9.26, and 9.71 μm. The y-polarized response is shown in Figure 3d. In this case, the difference between the baseline-calibrated spectrum and the biofilm-covered spectrum is much weaker. This result confirms the polarization-dependent behavior of the structure. The horizontal Au strips are efficiently excited by x-polarized light, while y-polarized light gives weak coupling.

**Figure 4:**
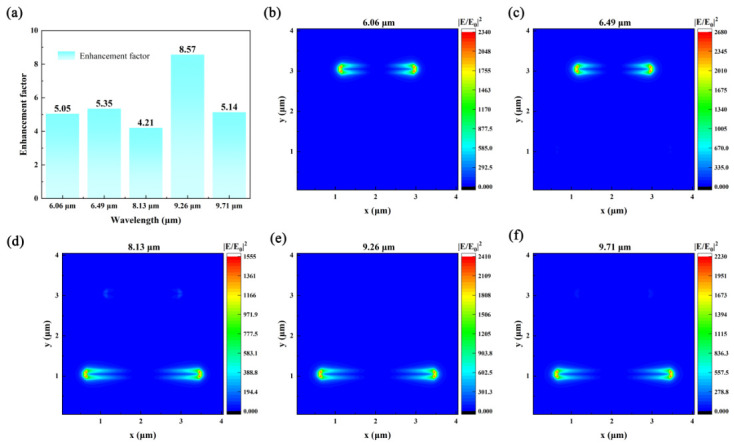
Enhancement factor and near-field distributions of the polarization-dependent metasurface. (**a**) Enhancement factors at selected biofilm absorption wavelengths. (**b**–**f**) Simulated |E/E0|^2^ distributions at 6.06, 6.49, 8.13, 9.26, and 9.71 μm.

To quantify the enhancement, the enhancement factor was defined as follows:

(6) $$\mathrm{EF}(\lambda )\;=\;\frac{A_{\mathrm{SEIRA}}\left(\lambda \right)}{A_{\mathrm{bare}}\left(\lambda \right)}$$

where Abare is the absorption of the bare biofilm without the metasurface. As shown in Figure 4a, the enhancement factors at 6.06, 6.49, 8.13, 9.26, and 9.71 μm are 5.05, 5.35, 4.21, 8.57, and 5.14, respectively. The largest enhancement factor appears at 9.26 μm. The near-field distributions are shown in Figure 4b–f. The normalized electric field intensity |E/E0|^2^ is mainly concentrated at the ends of the Au strips. These hot spots increase the local field around the biofilm region and enhance the interaction between the infrared field and the biofilm absorption. This explains the strong SEIRA response under x-polarized incidence.

## 5. Polarization-Insensitive SEIRA Metasurface

**Figure 5:**
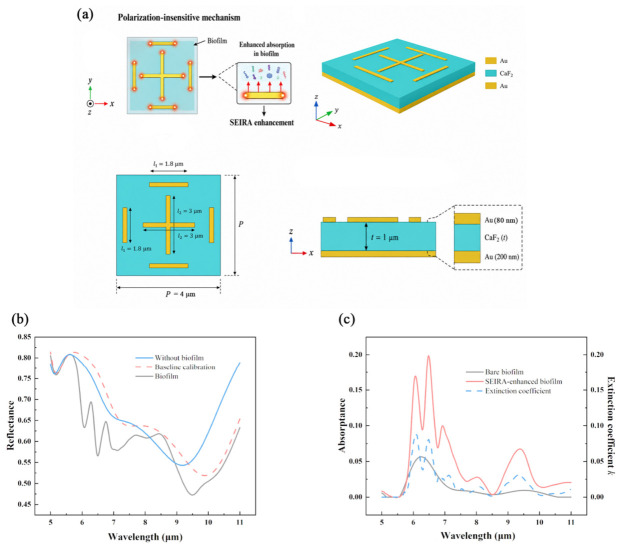
Polarization-insensitive metasurface and spectral response. (**a**) Schematic design of the polarization-insensitive metasurface. The red regions indicate the locations of strong electric-field oscillations in the resonator. The multicolored layer covering the metasurface represents the biofilm. (**b**) Reflectance spectra. (**c**) Bare and SEIRA-enhanced biofilm absorption spectra.

The second metasurface was designed as a polarization-insensitive structure. As shown in Figure 5a, the top resonator consists of a central cross and four surrounding short Au strips. The central cross length was set as l_2_ = 3 μm and the surrounding strip length was set as l_1_ = 1.8 μm. The unit period was P = 4 μm. The symmetric layout allows the structure to support similar resonant responses for x- and y-polarized incidence.

The reflectance spectra are shown in Figure 5b. The spectra include the metasurface without biofilm, the baseline calibration, and the metasurface with biofilm. Compared with the polarization-dependent structure, this design provides a more balanced response for the two orthogonal polarizations. After baseline calibration, the SEIRA-enhanced absorption spectrum was obtained using the same method as in Section 4.

The enhanced absorption spectrum is shown in Figure 5c. Clear absorption features can be observed near 6.06, 6.49, 8.13, 9.26, and 9.71 μm. These peaks agree with the main absorption bands of the bare biofilm and the extracted extinction coefficient. This result shows that the polarization-insensitive structure can enhance the infrared absorption response of the biofilm without requiring a fixed incident polarization.

**Figure 6:**
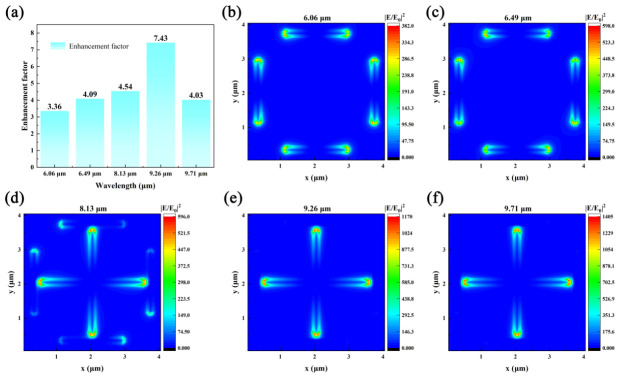
Enhancement factor and near-field distributions of the polarization-insensitive metasurface. (**a**) Enhancement factors at selected biofilm absorption wavelengths. (**b**–**f**) Simulated |E/E0|^2^ distributions at 6.06, 6.49, 8.13, 9.26, and 9.71 μm.

The enhancement factors are shown in Figure 6a. At 6.06, 6.49, 8.13, 9.26, and 9.71 μm, the enhancement factors are 5.10, 5.33, 3.85, 7.24, and 4.01, respectively. The highest value appears to be 9.26 μm.

The near-field distributions at the five selected wavelengths are shown in Figure 6b–f. The field enhancement is mainly located at the ends of the Au strips and near the edges of the central cross. These localized fields increase the interaction between the infrared field and the biofilm layer. Since the resonator has a symmetric geometry, the enhanced response can be maintained for different in-plane polarization directions. The vertical near-field distribution was also examined, and the enhanced field was mainly confined within approximately 150 nm above the resonator surface (Figure S4).

To further compare the present design with reported infrared-enhancement studies for bacteria or biofilms, representative works are summarized in Table 1.

Compared with previous studies, the present work focuses on multi-band mid-infrared fingerprint enhancement of experimentally parameterized *S. aureus* biofilm and provides both polarization-dependent and polarization-insensitive metasurface designs.

## 6. Robustness Analysis and Limitations

**Figure 7:**
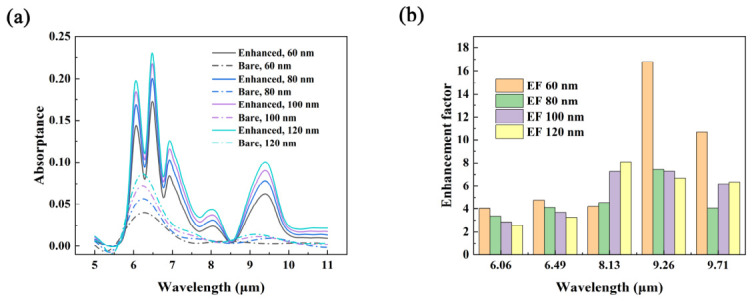
Thickness-dependent response of the polarization-insensitive metasurface. (**a**) Bare and SEIRA-enhanced absorption spectra for biofilm thicknesses of 60, 80, 100, and 120 nm. (**b**) Corresponding enhancement factors at the selected fingerprint wavelengths.

The effect of biofilm thickness was further evaluated using the polarization-insensitive metasurface. As shown in Figure 7a, the main absorption features were maintained when the effective biofilm thickness varied from 60 to 120 nm. The absolute absorption intensity increased with thickness, while the enhancement factor showed wavelength-dependent variations (Figure 7b). Enhanced responses were still obtained at all selected fingerprint wavelengths, indicating that the proposed design is not limited to the single 80 nm thickness assumption. To further examine the influence of the effective thickness used for extracting k, additional calculations were performed using k values recalculated from assumed thicknesses of 100 and 150 nm. As shown in Figure S1, the enhanced fingerprint features are still maintained, and the highest enhancement remains near 9.26 μm, indicating that the main enhancement trend is not solely dependent on the original 80 nm assumption.

**Figure 8:**
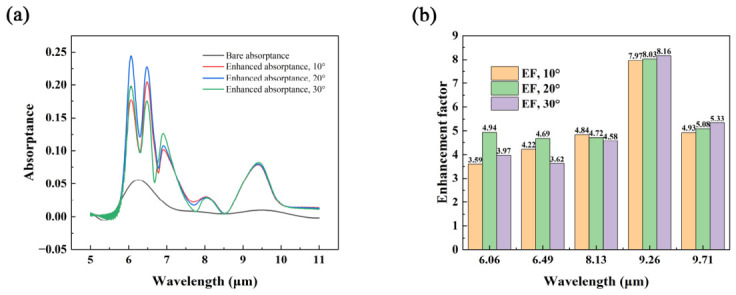
Oblique-incidence response of the polarization-insensitive metasurface. (**a**) SEIRA-enhanced absorptance spectra under incidence angles of 10°, 20°, and 30°, compared with the bare biofilm absorptance under normal incidence. (**b**) Corresponding enhancement factors at the selected fingerprint wavelengths.

Oblique-incidence simulations were further performed for the polarization-insensitive metasurface. In these simulations, the BFAST source was used, and the boundary conditions in the x and y directions were changed to Bloch boundary conditions. As shown in Figure 8a, the enhanced absorptance spectra under incidence angles of 10°, 20°, and 30° still retain the main biofilm fingerprint features. The corresponding enhancement factors are shown in Figure 8b. Clear enhancement is maintained at all selected wavelengths, and the highest enhancement remains near 9.26 μm, indicating that the proposed structure has a certain tolerance to moderate oblique incidence.

A limitation of the present work is that the proposed metasurface has not yet been fabricated and experimentally validated. In practice, the MIM structure could be fabricated by depositing a bottom Au layer, a CaF_2_ spacer layer, and patterned top Au resonators using electron-beam lithography followed by metal deposition and lift-off. Because the resonance position is closely related to the resonator dimensions, fabrication errors may affect the spectral matching between the metasurface resonance and the biofilm absorption bands. Therefore, a line-length tolerance analysis was performed by varying the Au resonator length by ±10%. As shown in Figure S3, the enhancement factors change with the dimensional deviation, but clear enhancement is still maintained at the selected fingerprint wavelengths. This result suggests that the design has a certain tolerance to line-length errors, although accurate nanofabrication remains important for maintaining optimal SEIRA performance. It should also be noted that the selected infrared bands are associated with general biochemical components, such as proteins, carbohydrates, and phosphate-related groups, and are not unique to *S. aureus*. Therefore, the present work does not demonstrate species-specific identification. Instead, it focuses on enhancing weak mid-infrared fingerprint signals from *S. aureus* biofilms. Future work should combine enhanced spectra with multi-species controls and statistical or machine-learning analysis to evaluate selectivity.

## 7. Conclusions

In this work, a metasurface-enhanced mid-infrared biosensor was designed to enhance the mid-infrared fingerprint response of *Staphylococcus aureus* biofilms. The optical response of the biofilm was first obtained from FTIR transmission measurements and AFM-based thickness characterization. The extracted extinction coefficient was then used to build an effective optical model for the following simulations. A metal–insulator–metal metasurface was optimized to match the main absorption bands of the biofilm. The parameter scan showed that the resonance position can be tuned by the Au resonator length and the CaF_2_ spacer thickness. A spacer thickness of 1 μm was selected to obtain a broad resonance with moderate absorption, which provides room for resolving the biofilm-induced absorption signal. Two metasurface designs were studied: one polarization-dependent structure and one polarization-insensitive structure. Both structures enhanced the main fingerprint bands of the biofilm. The maximum enhancement factors reached 8.57 for the polarization-dependent structure and 7.24 for the polarization-insensitive structure, both appearing near 9.26 μm. The near-field maps showed that the enhanced response mainly came from localized electric-field hot spots near the metal resonator edges. Additional robustness analyses showed that the main enhancement trend was maintained under variations in effective biofilm thickness, moderate oblique incidence, polarization angle, and line-length tolerance, although experimental fabrication and validation remain necessary for practical sensing applications. These results provide a practical design approach for enhancing multiple mid-infrared fingerprint bands of *S. aureus* biofilm.

## Figures and Tables

**Table 1:** Comparison with representative infrared-enhancement studies for bacteria or biofilms.

Study	Target	Working Region	Enhancement Factor	Polarization Dependence
Ref. [17]	*E. coli*, *S. aureus*, *B. subtilis*	Mid-infrared fingerprint region	Not directly reported as a unified EF	Not discussed
Ref. [18]	Microorganisms including *S. aureus*	Mid-infrared fingerprint region	On the order of 10	Not discussed
Ref. [19]	*S. aureus* bacterial films	Infrared absorption microscopy region	Surface-dependent enhancement	Not discussed
This work	*S. aureus* biofilm	5–11 μm	8.57/7.24	Polarization-dependent and polarization-insensitive designs

## Data Availability

The data supporting the findings of this study are available from the corresponding author upon reasonable request.

## Supplementary Materials

The following supporting information can be downloaded at https://www.mdpi.com/article/10.3390/bios16070397/s1.

## Abbreviations

The following abbreviations are used in this manuscript:

SEIRA	Surface-enhanced infrared absorption
FTIR	Fourier-transform infrared spectroscopy
AFM	Atomic force microscopy
FDTD	Finite-difference time-domain method
