# Supplementary material for: Design of a Metasurface-Enhanced Mid-Infrared Biosensor for Fingerprint Signal Enhancement of Staphylococcus aureus Biofilms
Source: Biosensors (Basel). 2026 Jul 22;16(7):397. doi: 10.3390/bios16070397 (PMC13406296; doi:10.3390/bios16070397)
Supplement: Supplementary file 1 [file biosensors-16-00397-s001.zip › biosensors-4374401-supplementary.pdf]

Supplementary Materials

The SEIRA response was recalculated using extinction coefficients derived from alternative effective biofilm thicknesses of 100 and 150 nm. In both cases, the enhanced absorptance spectra retain the main fingerprint features, and clear enhancement is obtained at the selected wavelengths. The enhancement factors vary with the assumed thickness, but the dominant enhancement near 9.26 μm is preserved.

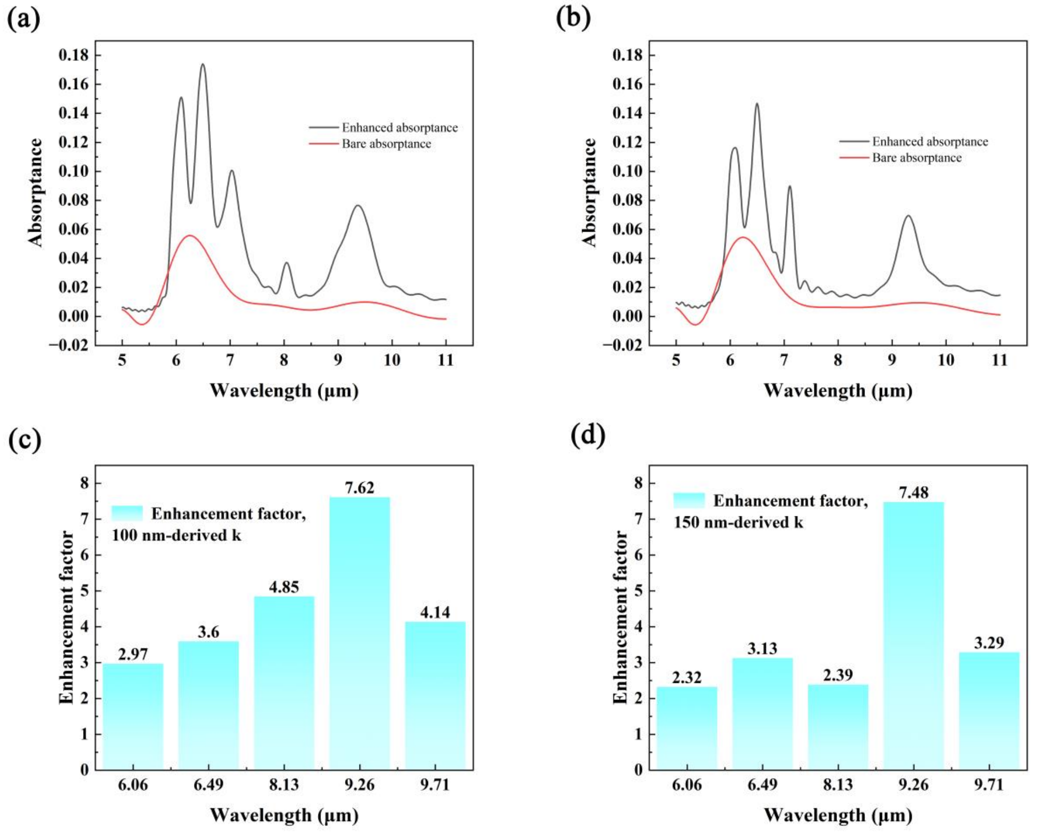

Figure S1. SEIRA response calculated using k values derived from alternative effective biofilm thicknesses. (a,b) Bare and enhanced absorptance spectra calculated using 100 nm-derived k and 150 nm-derived k, respectively. (c,d) Corresponding enhancement factors at the selected fingerprint wavelengths.

The polarization-angle dependence of the polarization-insensitive metasurface was evaluated from 0° to 90°. The reflectance map in Fig. S2 shows nearly unchanged spectral features over the full polarization-angle range, supporting the polarization-insensitive behavior of the design.

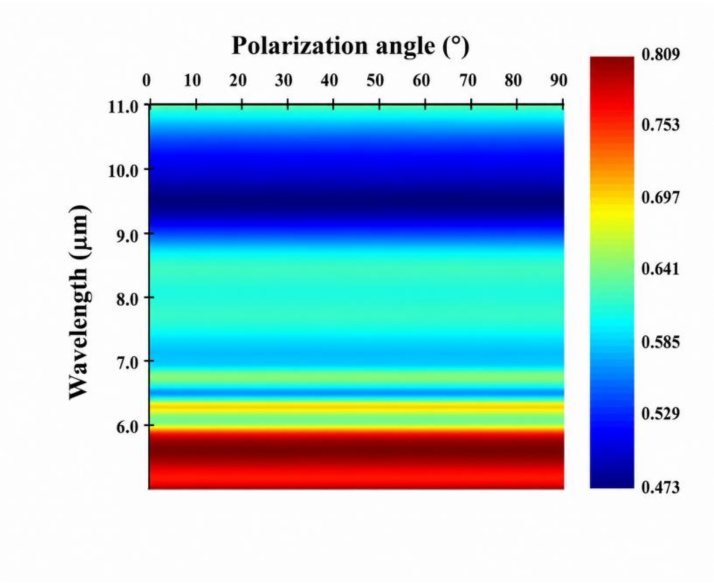

Figure S2. Reflectance map of the polarization-insensitive metasurface with biofilm as a function of wavelength and polarization angle.

The influence of fabrication tolerance was further investigated by changing the Au line length by ±10%. As shown in Fig. S3, the enhanced absorptance spectra and enhancement factors are affected by the line-length variation, but clear enhancement is still maintained at the selected fingerprint wavelengths. These results suggest that line-length errors can influence the absolute enhancement strength, while the main enhancement trend remains robust.

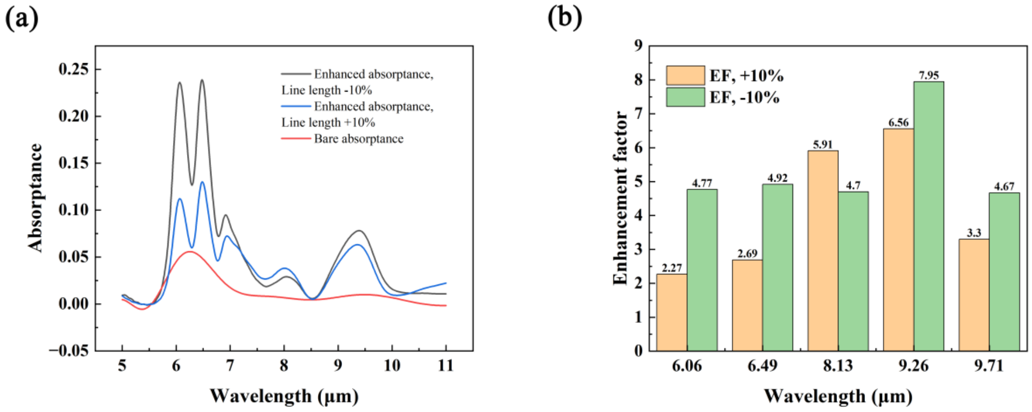

Figure S3. Influence of line-length tolerance on the SEIRA response. (a) Bare and enhanced absorptance spectra for line-length variations of +10% and −10%. (b) Corresponding enhancement factors at the selected fingerprint wavelengths.

The vertical electric-field distribution of the polarization-insensitive metasurface was further calculated to evaluate the near-field extension above the resonator surface. As shown in Fig. S4, the enhanced field is mainly localized near the Au resonator edges and extends into the region above the metasurface surface, indicating that biofilm components located close to the surface can effectively interact with the enhanced infrared field.

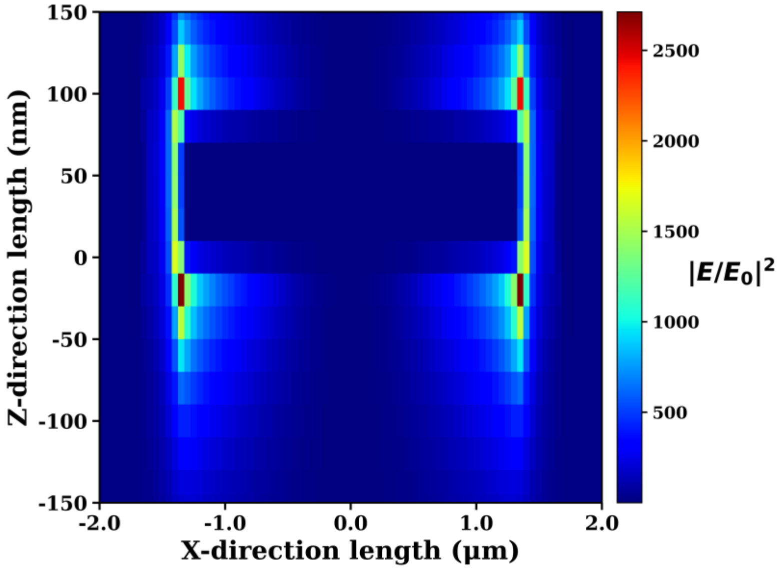

Figure S4. Simulated vertical electric-field distribution of the polarization-insensitive metasurface. The color scale represents the normalized electric-field intensity $|E/E0|^2$.
